# Studying the Effects of Granulocyte-Macrophage Colony-Stimulating Factor on Fetal Lung Macrophages During the Perinatal Period Using the Mouse Model

**DOI:** 10.3389/fped.2021.614209

**Published:** 2021-03-11

**Authors:** Fook-Choe Cheah, Pietro Presicce, Tian-Lee Tan, Brenna C. Carey, Suhas G. Kallapur

**Affiliations:** ^1^Neonatal Intensive Care Unit, Department of Paediatrics, Faculty of Medicine, Universiti Kebangsaan Malaysia Medical Centre, Hospital Canselor Tuanku Muhriz, Kuala Lumpur, Malaysia; ^2^Division of Pulmonary Biology, Cincinnati Children's Hospital Medical Center, Cincinnati, OH, United States; ^3^David Geffen School of Medicine, University of California, Los Angeles, Los Angeles, CA, United States

**Keywords:** bronchopulmonary dysplasia, chorioamnionitis, GM-CSF overexpression, intrauterine inflammation or infection, GM-CSF knockout, interstitial macrophages, alveolar macrophages, transgenic mice

## Abstract

**Background:** Granulocyte-macrophage colony-stimulating factor (GM-CSF) is a pro-inflammatory cytokine that is increased in the amniotic fluid in chorioamnionitis and elevated in the fetal lung with endotoxin exposure. Although GM-CSF has a pivotal role in fetal lung development, it stimulates pulmonary macrophages and is associated with the development of bronchopulmonary dysplasia (BPD). How antenatal GM-CSF results in recruitment of lung macrophage leading to BPD needs further elucidation. Hence, we used a transgenic and knock-out mouse model to study the effects of GM-CSF focusing on the fetal lung macrophage.

**Methods:** Using bitransgenic (BTg) mice that conditionally over-expressed pulmonary GM-CSF after doxycycline treatment, and GM-CSF knock-out (KO) mice with no GM-CSF expression, we compared the ontogeny and immunophenotype of lung macrophages in BTg, KO and control mice at various prenatal and postnatal time points using flow cytometry and immunohistology.

**Results:** During fetal life, compared to controls, BTg mice over-expressing pulmonary GM-CSF had increased numbers of lung macrophages that were CD68^+^ and these were primarily located in the interstitium rather than alveolar spaces. The lung macrophages that accumulated were predominantly CD11b^+^F4/80^+^ indicating immature macrophages. Conversely, lung macrophages although markedly reduced, were still present in GM-CSF KO mice.

**Conclusion:** Increased exposure to GM-CSF antenatally, resulted in accumulation of immature macrophages in the fetal lung interstitium. Absence of GM-CSF did not abrogate but delayed the transitioning of interstitial macrophages. Together, these results suggest that other perinatal factors may be involved in modulating the maturation of alveolar macrophages in the developing fetal lung.

## Introduction

Granulocyte-macrophage colony-stimulating factor (GM-CSF) is a glycoprotein which was discovered as a hemopoietic growth factor based on its ability to stimulate bone marrow cell proliferation in a mouse lung-conditioned medium (1). It is now evident that GM-CSF also exhibits immunomodulatory effects and serves as one of the major cytokines during inflammation (2–4). In the lung, the locally expressed GM-CSF have both autocrine and paracrine functions on type II alveolar epithelial cells and alveolar macrophages, respectively (5, 6). During pregnancy, the level of GM-CSF is developmentally regulated and is elevated in the presence of chorioamnionitis and other inflammatory conditions (7, 8). Endotoxin-induced chorioamnionitis resulted in increased GM-CSF in the amniotic fluid and fetal sheep lung, but not in the fetal circulation, suggesting that chorioamnionitis induced local expression of GM-CSF and could have profound effects on the developing fetal lung (9).

Intriguingly, augmentation of GM-CSF resulted in the proliferation of type II alveolar epithelial cells, restoration of alveolar epithelial barrier function, increased vascular endothelial growth factor expression, enhancement of lung growth and surfactant phospholipid production in several animal models, suggesting the role of this cytokine in the lung reparative and remodeling process (5, 10–12). These may underlie clinical observations of a lesser burden of respiratory distress syndrome in preterm infants with intrauterine exposure to chorioamnionitis (13–15). In fact, GM-CSF is essential for alveolar macrophage (AM) phenotypic and functional maturation via its downstream transcriptional factor PU.1 and peroxisome proliferator-activated receptor-γ (PPAR-γ) (16–19). As the AM is also crucial in alveolar surfactant catabolism (16, 17), ablation of the mice genetic loci for GM-CSF, although does not perturb lung morphogenesis (10), leads to the abnormal deposition of surfactant in the alveolar space, a typical characteristic of pulmonary alveolar proteinosis (16, 20).

The development of fetal AM is thought to begin antenatally in the lung interstitium and translocate into the alveolar sacs in the first week of postnatal life (18, 21–24). The fetal monocytes first colonize the developing lung and differentiate into the primitive AM from embryonic day 18.5 in the lung interstitium, which corresponds to the saccular phase of alveolar development (18). Subsequently, the primitive AM differentiates into mature AM under the influence of GM-CSF surge that occurs in the perinatal period, and they reside in the alveoli with minimal turnover (18). However, more recently, the study on the ontogeny of lung macrophages elegantly showed that during postnatal and adult life, the lung interstitium could be re-populated in a third “wave” with non-embryonic bone marrow-derived macrophages (25). This phase may be less dependent on GM-CSF as Dranoff et al. in their seminal paper surmised that the ablation of this cytokine did not appear to critically affect basal hematopoesis (26).

During an inflammatory process, macrophage recruitment in the alveolar sacs are associated with delayed alveolar and pulmonary vasculature development, similar to lung changes seen in infants with bronchopulmonary dysplasia (BPD) (27–29). In preterm infants, the early alteration in pulmonary expression of GM-CSF during postnatal life has been found to precede the development of BPD (30).

Together, these observations suggest a balance in GM-CSF levels may exist during the perinatal period affecting AM trafficking and maturation, which is still largely unclear. Disruption of this balance may predispose to the development of BPD in preterm infants. Therefore, we used animal models with GM-CSF over-expression and knock-out to study the effects on the AM during the saccular phase of alveolar development in late gestation and early postnatal life. The results may serve as a platform for the further evaluation of the direct effects of GM-CSF on BPD development.

## Materials and Methods

### Mice

Animal experiments were performed following protocols approved by the Cincinnati Children's Hospital Medical Center Animal Care Committee (IACUC S3D01004). Wild type mice (Charles River, Wilmington MA), and transgenic mice in this study were on the C57Bl/6 background. All mice were housed in a barrier facility with a continuous supply of purified water, air and food, which was supplemented with doxycycline (Dox) if indicated. The female mice were checked for vaginal plug the morning after the night of breeding, and plug presence was recorded as embryonic day 0.5 (E0.5). For the study of fetal lungs, the pregnant dams were euthanized by carbon dioxide narcosis, the fetuses surgically delivered and mouse pups were sacrificed with intraperitoneal pentobarbital injection.

### GM-CSF Overexpressing and GM-CSF Knockout Mice

Inducible lung-specific GM-CSF expressing bi-transgenic mice (BTg) were generated previously by crossing the reverse tetracycline-activator (rtTA)-Clara cell secretory protein (CCSP) line with the (tet-O)_7_-CMV-GM-CSF line. In this system, inducible expression of GM-CSF is mediated by rtTA binding to the tet-operator, switched on by Dox administered in the food. Lung epithelial-specific GM-CSF expression is mediated by the CCSP promoter, which directs transgene expression at high levels in non-ciliated respiratory epithelial cells of the tracheal-bronchial and bronchiolar regions of the lung starting at embryonic day 14.5 (31). To generate single transgenic (STg) mice [rtTA-CCSP or (tet-O)_7_-CMV-GM-CSF] and WT mice as littermate controls for BTg mice, male BTg mice were bred with WT females. Pregnant mice received daily Dox starting at gestational day E14.5. The GM-CSF KO mice, generated by Dr. Glenn Dranoff (26), were backcrossed for at least eight generations into the C57Bl/6 strain.

### Genotyping

Mouse tails were digested using proteinase K (1 mg/mL) in lysis buffer containing Tris HCL pH 8, 0.5 M EDTA, 5 M NaCl, and 20% SDS. The samples were precipitated with KOAc and washed with pure ETOH followed by 70% ETOH, dried, and the pellets were reconstituted in Tris-EDTA buffer. Genotyping was done using the following primers with GoTaq Green Master Mix (Promega Corporation, Madison, WI) with minor modifications of the manufacturer's protocol.

CCSP-rtTA 5′ ACT GCC CAT TGC CCA AAC AC
CCSP-rtTA 3′ AAA ATC TTG CCA GCT TTC CCC
(tet-O)_7_-GM-CSF 5′ GCC ATC CAC GCT GTT TTG AC
(tet-O)_7_-GM-CSF 3′ CCT GGG CTT CCT CAT TTT TGG.

### Animal Procedures

To obtain bronchoalveolar lavage fluid (BALF), the fetal (E18.5) and neonatal (postnatal, PN day 1–14) mice were dissected under a microscope, trachea exposed and cannulated and flushed with 0.5–1.0 mL cold sterile PBS (pH 7.4) through a 25 Fr gauge needle. BALF was used for cell counts and differential counts using Diff-Quik staining, flow cytometry cell studies and for GM-CSF protein measurement in the supernatant collected from select animals using ELISA assay (Endogen, Inc., Boston, MA). Lung cell suspensions were made in tissue culture media (RPMI + 10% FCS) by mincing the left lung under gentle pressure and filtered using a 50 μm nylon mesh.

### Flow Cytometric Analyses

Lung cells were suspended at a density of 1 × 10^6^ cells/100 μL media. Cell viability was determined using trypan blue exclusion and was found to be >90% viable for all the experiments. The antibodies used in the study were based on validated markers for immunophenotyping lung monocyte/macrophage (32). The following antibodies were used: Rat anti-mouse F4/80 - FITC (BM8) (Abcam, Cambridge, MA), CD11b - APC (M1/70) (BD Pharmingen, San Diego, CA) and CD68 - RPE (AbD Serotec, Raleigh, NC). Appropriate isotype-control antibodies were used. The cells were reacted with the antibodies for 30 min on ice followed by washing × 3. At least 10,000 events were recorded for each sample using the BD FACSCalibur™ flow cytometer (Becton, Dickinson and Co., Franklin Lakes, NJ), and analyses were done using the FlowJo software (Ver 9.5.2, Treestar, Ashland, OR). Cells above the isotype background intensity were considered as positively stained cells. The values were expressed as proportions of positively labeled cells.

### Lung Tissue Immunohistochemistry

For lung tissue immunohistochemistry, thoraces from embryonic and PN day 1 pups were immersed in ice-cold 4% paraformaldehyde in PBS overnight. For PN day 4, 7, and 14 mouse pups, after cannulation of the trachea, the lungs were inflation fixed with 4% paraformaldehyde at 25 cm H_2_O pressure. The fixed lung tissues were washed three times in PBS and dehydrated in ethanol before paraffin embedding or immersed in 30% sucrose followed by mounting in the cryoprotectant OCT and frozen at −80°C before immunostaining. Lung tissues for CD68 were immunostained using 5 μm sections from OCT embedded lung specimen (Rat anti-mouse CD68 AbD Serotec, Raleigh, NC). Myeloperoxidase (MPO) staining of lung samples was done on formalin fixed lung tissue cut in 5 μm sections (rabbit polyclonal anti-MPO Cell Marque Corp., Rocklin, CA). For the paraffin embedded sections, antigen retrieval was performed by boiling the tissues in citrate buffer followed by quenching of endogenous peroxidase activity with hydrogen peroxide. Non-specific binding was blocked with 5% goat serum before overnight incubation at 4°C with anti-CD68 (1:100) or anti-MPO antibody (1:200). The sections were washed followed by incubation at room temperature for 1 h with a secondary biotinylated goat anti-rabbit antibody (Vector Laboratories, Burlingame, CA) diluted at 1:200. Immunoreactivity was determined by the diaminobenzidine method. The tissues were counterstained with nuclear fast red. Quantitative cell counts were performed in a blinded fashion by counting CD68^+^ cells in 5 random microscopic fields per animal in at least 3 animals per group.

### Statistics

Statistical analyses were performed with SigmaStat v.1.0 (Jandel, San Rafael, CA) and GraphPad, Prism v.6.03 (GraphPad Software, Inc., La Jolla, CA). Comparisons of two groups were made with unpaired *t*-tests and the results are presented as means and SD. For comparisons of more than two groups, ANOVA followed by Student-Newman-Keuls tests for *post-hoc* analyses were used. Significance was accepted at *P* < 0.05.

## Results

We used both WT and STg littermates as controls for the BTg experimental fetal mice. We confirmed the non-inducible GM-CSF expression in the WT and STg littermates showing no differences in the percentages of positive F4/80 and CD11b cells on flow cytometry or CD68 positive macrophages on lung immunohistology after Dox treatment at E16.5 and E18.5 (Supplementary Figure 1). Thus, the controls used in this study were a composite of WT and/or STg genotypes. Similarly, in the CCSP-rtTA model with constitutive and inducible *Fgf*-7, WT and STg did not overexpress when given Dox as compared with the BTg (31).

### Antenatal GM-CSF Overexpression Increased Macrophages in the Fetal Lung

**Figure 1:**
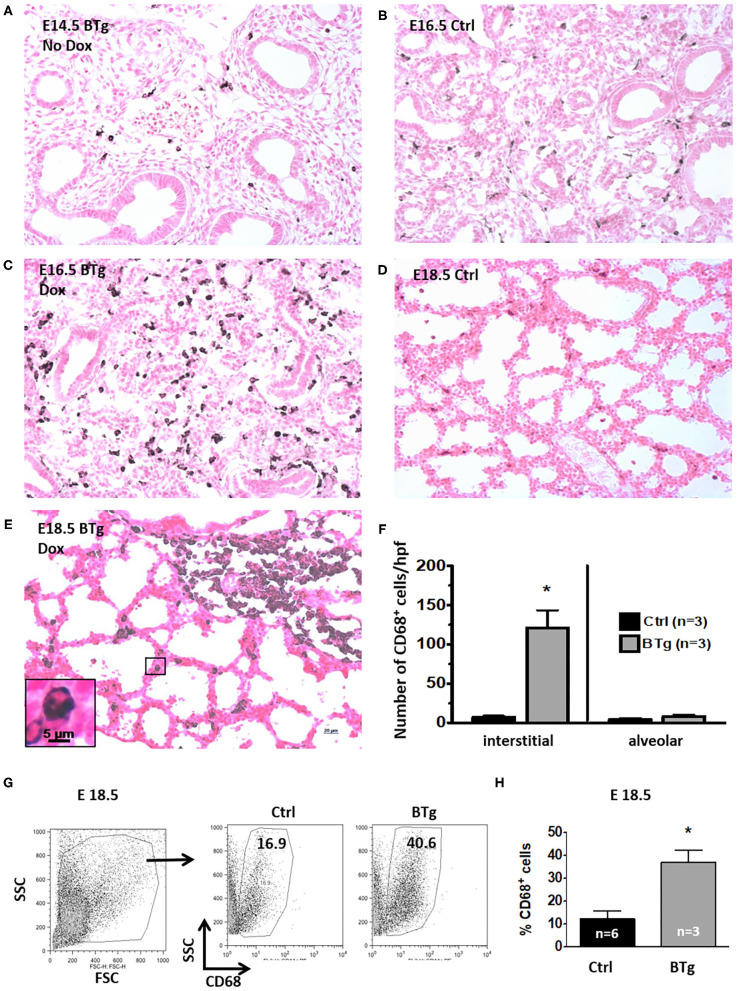
Antenatal GM-CSF increased interstitial CD68^+^ cells in the fetal lung. Representative immunohistology for CD68 using lung sections (20X objective) from **(A)** Bitransgenic (BTg) mice at E14.5 without exposure to doxycycline (Dox), **(B)** control E16.5 and **(D)** control E18.5 fetuses. Sections from BTg fetuses after exposure to Dox at **(C)** E16.5 and **(E)** E18.5. Inset in **(E)** shows a magnification of a CD68^+^ cell in the interstitium with the characteristic mononuclear morphology. Increased staining for CD68 (dark brown/black staining) expressing cells were noted in the interstitium of BTg mice at E16.5 and E18.5. **(F)** Quantification of interstitial vs. alveolar location of CD68^+^ cells (by immunohistology) in E18.5 BTg fetuses. **(G,H)** Flow cytometry quantification using E18.5 fetuses shows more than two-fold increase in CD68^+^ cells in BTg fetal lungs compared to control lungs (*p < 0.05 vs. controls).

Switching on with Dox resulted in markedly increased GM-CSF in BALF of BTg, E18.5 fetal mice (236 ± 171 pg/mL for BTg mice vs. 2.5 ± 2.8 pg/mL, for controls, respectively, *p* < 0.05. There was no difference in mean GM-CSF levels between the WT and STg; 3.2 and 2.3 pg/mL, respectively). This was consistent with a previously reported study that constitutive levels of GM-CSF in the BALF of these mice were at the limit of detection of the assay, 5 pg/mL (10). Concordantly, compared to littermate controls, BTg fetuses exposed to Dox at E14.5 showed accumulation of CD68^+^ macrophages by immunohistology on days E16.5 and E18.5 (Figures 1A–E). These cells had a typical mononuclear morphology (Figure 1E, inset), and lacked MPO expression. Almost all the lung macrophages in the E18.5 BTg mice were interstitial with <5% alveolar macrophages (Figure 1F). Immuno-phenotyping results of these interstitial cells by flow cytometry of fetal lung suspensions in response to GM-CSF is shown in Figure 1G. Compared to control littermates, BTg fetuses had a 3-fold increase in lung CD68^+^ cells (Figure 1H).

**Figure 2:**
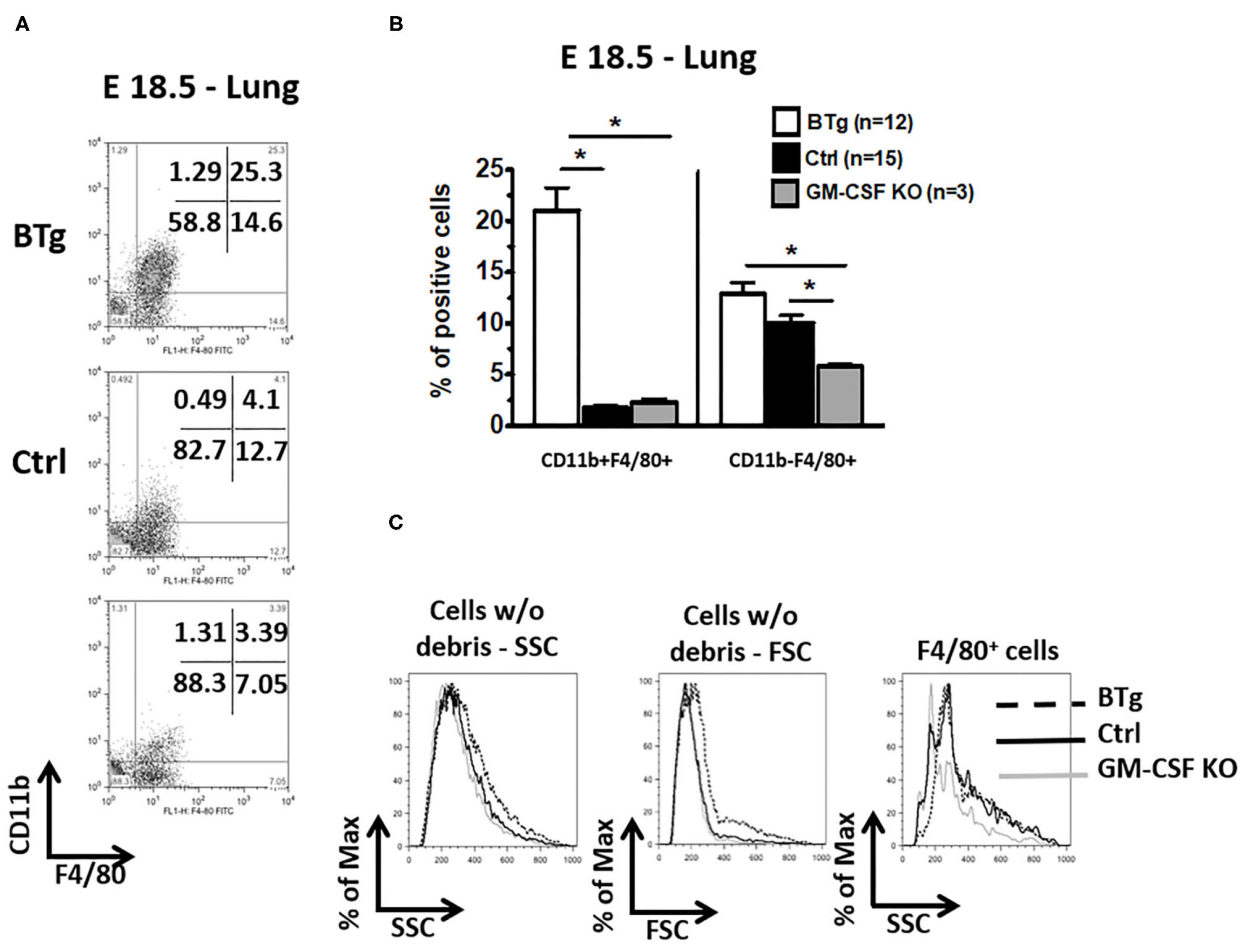
Increased double positive CD11b/F4/80 (CD11b^+^ F4/80^+^) fetal lung macrophages after antenatal GM-CSF exposure. Lung cell suspensions were made from fetuses at E18.5 with the genotypes BTg on Dox starting at E14.5, GM-CSF KO, and controls. Cell suspensions were labeled with anti-CD11b and anti-F4/80 for flow cytometry. **(A)** Representative dot plots, **(C)** the corresponding quantitation for the gated cells in **(A)** are shown, **(B)** histogram showing scatter properties of the lung cells from the three groups. The major lung macrophage population in the BTg fetuses was CD11b^+^ F4/80^+^ (*p < 0.05 vs. controls).

It has been reported that interstitial lung macrophages are CD11b^+^ and F4/80^low^ while alveolar macrophages are CD11b^−^ and F4/80^high^ in adult mice (32). Moreover, immature macrophages have lower side and forward scatter properties (18). We therefore sought to further determine the characteristics of antenatal GM-CSF driven accumulation of macrophages. In control fetuses at E18.5, the alveolar phenotype CD11b^−^ F4/80^+^ macrophages were 5-fold more abundant compared to CD11b^+^ F4/80^+^ macrophages (Figures 2A,B). In contrast, the predominant population of macrophages in BTg fetuses at the similar timepoint showed predominantly the interstitial type CD11b^+^ F4/80^+^ macrophages. The alveolar:interstitial (CD11b^−^:CD11b^+^) macrophage ratio showed a reversal in phenotype with fewer alveolar but more interstitial at 1:2 ratio in BTg fetuses compared to 5:1 in controls. Finally, GM-CSF KO at E18.5 had cells with lower side and forward scatter properties compared to BTg fetuses (Figure 2C). These KO fetuses had the lowest alveolar type CD11b^−^ F4/80^+^ macrophages compared to both controls and BTg fetuses (Figure 2B).

### Fetal Lung Interstitial Macrophages Are Reduced but Not Absent in GM-CSF KO Mice

**Figure 3:**
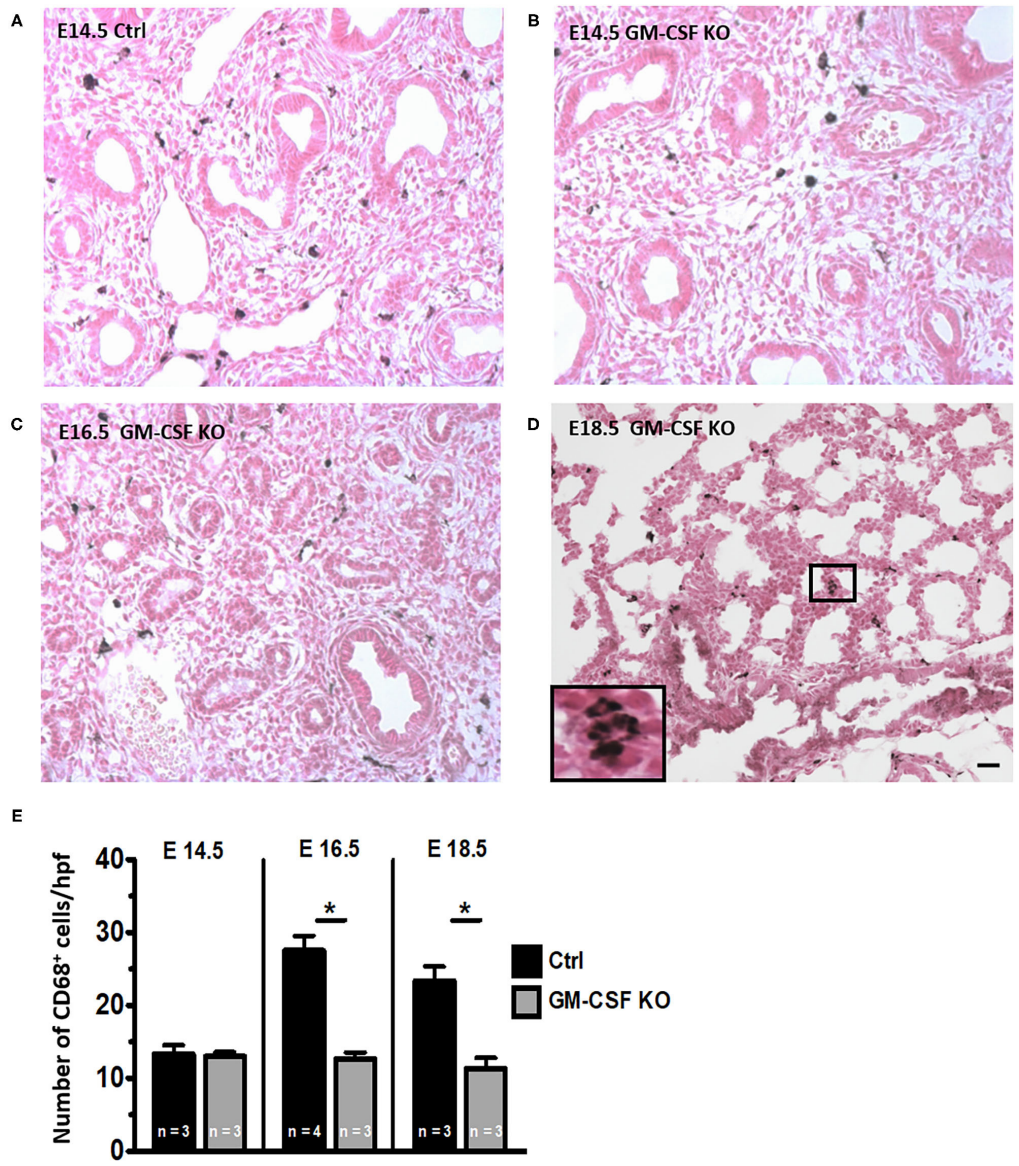
Decreased CD68^+^ macrophages in fetal lungs of GM-CSF KO fetuses. Representative immunohistology for CD68 using lung sections (20X objective) from **(A)** Control fetal lung at E14.5. **(B–D)** Fetal lungs from GM-CSF KO mice at the indicated gestational ages. Inset in **(D)** shows magnification of a group of interstitial CD68 staining macrophages with their characteristic morphology. **(E)** Quantitation of CD68^+^ cells based on the average number of cells present in at least five random high power fields (hpf) is shown. The number of macrophages was lower in GM-CSF KO compared to control lungs at E16.5 and E18.5. (*p < 0.05 vs. controls).

We next investigated whether GM-CSF was essential for the development of lung macrophages. At E14.5 there were very few CD68^+^ cells in the lung interstitium of both control and GM-CSF KO mice but the CD68^+^ cell counts were similar between these groups (Figures 3A,B,E). In the controls, there was a progressive increase in CD68^+^ interstitial macrophages from E14.5 to E16.5 and E18.5 (Figure 3E, see also, Figures 1A,B,D). In contrast, the CD68^+^ cell counts did not increase from E14.5 to E18.5 in the GM-CSF KO mice (Figures 3B–E), and the proportions of these CD68^+^ cells were lower in the GM-CSF KO mice compared to the controls (Figure 3E). Consistently, there were no alveolar macrophages in the fetal lungs of controls and KO.

### Lung Interstitial Macrophages Maturing as Alveolar Type Postnatally May Be Dependent on GM-CSF

**Figure 4:**
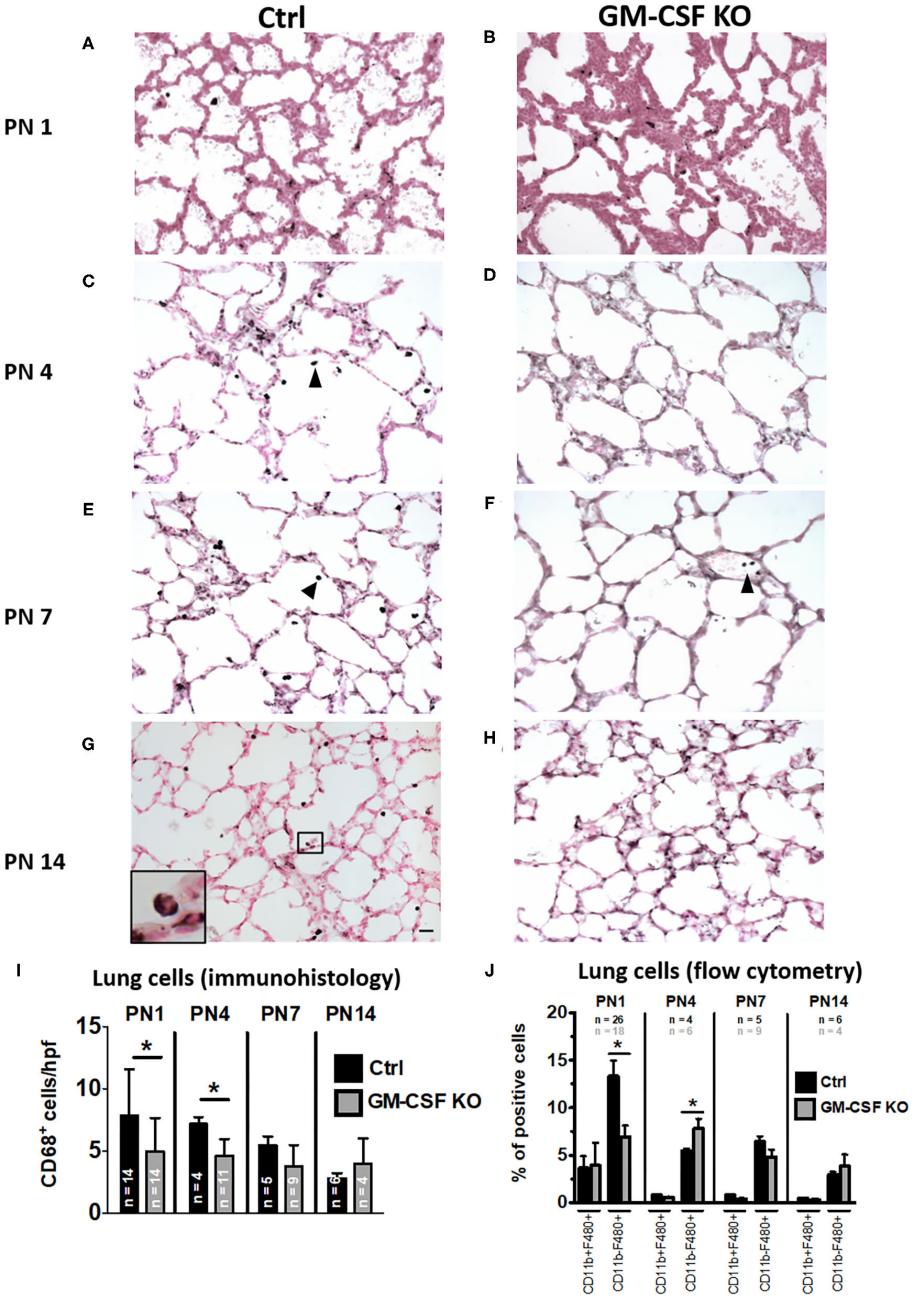
Decreased CD68^+^ macrophages in lungs of GM-CSF KO neonatal mice. Representative immunohistology of neonatal mouse lungs at postnatal (PN) days 1, 4, 7, and 14 (20X objective). **(A,C,E,G)** show immunostaining in control mice and **(B,D,F,H)** show immunostaining in GM-CSF KO mice. There were more alveolar macrophages (arrowhead showing dark brown/black staining cells) in the controls compared to the GM-CSF KO group. Inset in **(G)** shows a magnified CD68^+^ alveolar macrophage in control mice, but CD68^+^ cells were not seen in the alveolar space of GM-CSF KO mice (*N* = 4–13 animals/group). Immunohistology quantitation of the lung CD68^+^ cells comparing control and GM-CSF KO mice **(I)**. Flow cytometry quantitation of lung homogenates for proportions of cells positive for CD11b and/or F4/80 **(J)**. Differences in CD68^+^, CD11b^+^, and F4/80^+^ expression between controls and GM-CSF KO mice noted on PN1 and PN4 were no longer seen at PN7 and PN14 (*p < 0.05 vs. controls).

Absence of GM-CSF signaling causes disruption of alveolar macrophage function resulting in pulmonary alveolar proteinosis in an adult mouse model (16, 20). There is limited data to indicate if this phenomenon occurs even at the early neonatal period. To understand the postnatal ontogeny of lung macrophage and its dependence on GM-CSF, we compared immunohistology for CD68 in the lung tissue from GM-CSF KO vs. controls. As in the preceding antenatal period, despite the absence of GM-CSF in KO, CD68^+^ cells were still present in the lungs at postnatal days 1, 4, 7, and 14, although they appeared fewer than controls (Figure 4). Notably, on PN14, CD68^+^ cells were abundantly seen in the alveolar space of controls but not in the GM-CSF KO mice (compare Figures 4G,H). In the lung tissue, CD68^+^ cell numbers in the GM-CSF KO mice were decreased at PN1 and PN4 compared to controls, but were similar for PN7 and PN14 (Figure 4I). Similarly, CD11b^−^ F4/80^+^ macrophages were fewer in KO mice on PN1 than controls (Figure 4J). In BALF, a distinct population of cells from the alveolar space appeared with high forward and side scatter properties in the controls but this was completely absent in the KO mice (Supplementary Figures 2A,B). Consistent with immunohistology, KO mice also had fewer CD68^+^ cells (Supplementary Figure 2C) and CD11b^−^ F4/80^+^ macrophages in the BALF (Supplementary Figure 2D) than controls.

## Discussion

Our results demonstrate that antenatal surge of GM-CSF increased the population of CD68 and CD11b/F4/80 double-positive cells. These lung macrophages in the fetus just before birth were almost exclusively located in the interstitium rather than the alveoli. Although some loss of alveolar macrophages may occur during tissue processing, the overwhelming interstitial macrophage presence compared to controls was remarkably significant (Figure 1). Furthermore, compared with macrophages of postnatal day 14 mice, the lung macrophage from E18.5 fetuses had a significantly lower F4/80 expression and lower scatter properties, suggesting that antenatal GM-CSF increases the population of immature macrophages in the interstitium. A recent ontogeny study reported the three “waves” in lung macrophage development. The first two “waves” are “primitive” macrophages originating from the yolk sac and fetal liver, and they are located primarily in the lung interstitium with F4/80 and Mac2 (low F4/80) expression. These are gradually replaced by the postnatal form of “definitive” interstitial macrophages, also having F4/80 expression but presumably regulated by progenitors from bone-marrow dependent hematopoeisis (25). It is recognized that interstitial macrophages are involved in tissue repair and lung remodeling while alveolar macrophages are considered as the “police” of the alveolar space involved in phagocytosis of foreign particles and surfactant catabolism. The functional potential of interstitial macrophages is less clear, but macrophages from fetal mice can respond to LPS *in vitro* with the production of IL-1β and TNF-α (33). Our previous work in a preterm sheep model indicated that lung monocytes/macrophages from controls did not respond with oxidative burst to an inflammatory stimulus, but with prior intrauterine exposure to LPS it significantly increases the oxidative ability of these lung monocytes/macrophages (9). The functional competency of the interstitial lung macrophage induced by antenatal inflammation with cytokines such as GM-CSF surge *in vivo* awaits further studies. As GM-CSF levels have been reported to be increased in chorioamnionitis (7, 9, 34, 35), we speculate that these macrophages may be primed to a more robust response to extrauterine stresses such as hyperoxia and volutrauma from mechanical ventilation. As observed by Tan and Krasnow, physiologically, a population of interstitial macrophages (Mac2) began to populate the alveolar spaces at the end of the first postnatal week (25). Furthermore, a recent study showed that exposure to hyperoxia in the first 10 days postnatal life induced accumulation of macrophages in the alveolar space, possibly including a population of interstitial macrophages (36). We speculate the first “hit” from intrauterine infection upregulates GM-CSF and increases interstitial macrophages in the fetal lung. The second “hit” during postnatal life such as hyperoxia, triggers the release of these accumulated interstitial macrophages into the alveolar space. This “double-hit” hypothesis has been proposed by some authors in the pathogenesis of BPD (37, 38).

Even though the administration of GM-CSF may increase surfactant production in preterm rabbits (39), activated interstitial macrophages as the result of local GM-CSF surge impaired airway branching morphogenesis, which, together are some features of the new BPD (33). In a clinical study, GM-CSF levels were elevated in the tracheal aspirates of preterm infants who developed BPD (34). Together, tight regulation of GM-CSF levels appears to be necessary for normal fetal lung development and the maturation of alveolar macrophages.

Our study concludes that a surge of antenatal GM-CSF, as might happen in intrauterine inflammation, stimulates an increase in the interstitial macrophage population in the developing fetal lung. These GM-CSF-induced interstitial macrophages have the characteristics that are lacking of the mature alveolar type. Our results were consistent with what have been previously reported (18, 25). We further demonstrated that postnatally, GM-CSF is required for the early transition to this population of mature macrophages in the alveolar space. Also, the absence of GM-CSF showed a slower accumulation of CD68^+^ cells in the lung during the early postnatal period. Similarly, in the experiment by Kalymbetova et al. in depleting *Csf1r-*expressing monocytes or macrophages by Fas-induced apoptosis, few macrophages were still detected in the alveolar spaces (36). Collectively, our results suggest a novel finding that GM-CSF is necessary, but not the sole determinant for interstitial macrophage recruitment and alveolar macrophage maturation during the perinatal period. Other stimuli, perhaps the mechanical stretch from respiration postnatally, oxygen sensing or other cytokine signals, are likely needed for the accumulation of mature alveolar macrophages. Intuitively, interstitial macrophages and intrauterine factors may be the major modulators of the “new” BPD whereas alveolar macrophages may play a greater role in the more severe or “old” BPD (40).

## Conclusion

The Dox-inducible CCSP-promoter based transgenic mice model has been successfully developed for tight control of various gene expression to study lung development and disease pathogenesis. Concerns regarding toxicity of the rtTA system associated with emphysematous change in the lung structure of adult mice (41) was not evident at the perinatal or neonatal stages in our mouse model when comparing between the wild type and single transgene littermates. Of note, this transgenic model has rarely been used in studying antenatal inducible events followed by postnatal exposures to agents of interest.We hope our preliminary results and the models utilized here may serve as a platform to further study the mechanisms of disruption in the control and balance of GM-CSF during the perinatal period. How this could interfere with normal lung development and functional outcomes when complicated by postnatal factors such as hyperoxia and sepsis in the pathogenesis of BPD also warrant further studies.

## Data Availability Statement

The raw data supporting the conclusions of this article will be made available by the authors, without undue reservation.

## Ethics Statement

The animal study was reviewed and approved by Cincinnati Children's Hospital Medical Center Animal Care Committee (IACUC S3D01004).

## Author Contributions

F-CC and SGK were involved in designing of the study, analyses of the data, interpreting the results, and writing of this report. BCC was primarily involved with breeding of the animals, conducting the animal experiments, and data collection. PP was involved in the flow cytometry analyses, data interpretation, and reporting. T-LT was involved in analysis and writing of the manuscript. All authors contributed to the article and approved the submitted version.

## Conflict of Interest

The authors declare that the research was conducted in the absence of any commercial or financial relationships that could be construed as a potential conflict of interest.

## Supplementary Material

Supplementary Figure 1 Immunohistology and flow cytometry quantification of lung macrophages using CD68, F4/80 and CD11b markers in mice with constitutive and inducible GM-CSF backgrounds. **(A–E)** Represent results from E14.5 mice on regular chow and from mice who were on Dox at E16.5 and E18.5. There were no differences in CD68, F4/80 and CD11b positive macrophages between WT and STg at E14.5, E16.5 or E18.5. However, significantly more of these macrophages were present in BTg on Dox at E16.5 and E18.5. WT, wild type; STg, single transgenic; BTg, bitransgenic; ^*^*p* < 0.05 vs. controls.

Supplementary Figure 2 Immunophenotyping of BALF cells for monocyte/macrophage surface markers in control and GM-CSF KO neonatal mice. Flow cytometry was performed on BALF cells from control and GM-CSF KO neonatal mice at PN14. **(A)** Representative dot plots and **(B)** histogram of the forward-scatter (FSC) and side-scatter (SSC) properties of BALF cells. **(C)** Quantitation of CD68^+^ cells and **(D)** CD11b^+^ and F4/80^+^ cells. In contrast to the subtle differences between cell populations in lung homogenates (Figure 4J), BALF from GM-CSF KO mice had strikingly lower CD68^+^ cells, and lower CD11b^−^ F4/80^+^ cells compared to controls (^*^*p* < 0.05 vs. controls).
