# Supplementary figures and images for: Studying the Effects of Granulocyte-Macrophage Colony-Stimulating Factor on Fetal Lung Macrophages During the Perinatal Period Using the Mouse Model
Source: Front Pediatr. 2021 Mar 11;9:614209. doi: 10.3389/fped.2021.614209 (PMC7991795; doi:10.3389/fped.2021.614209)

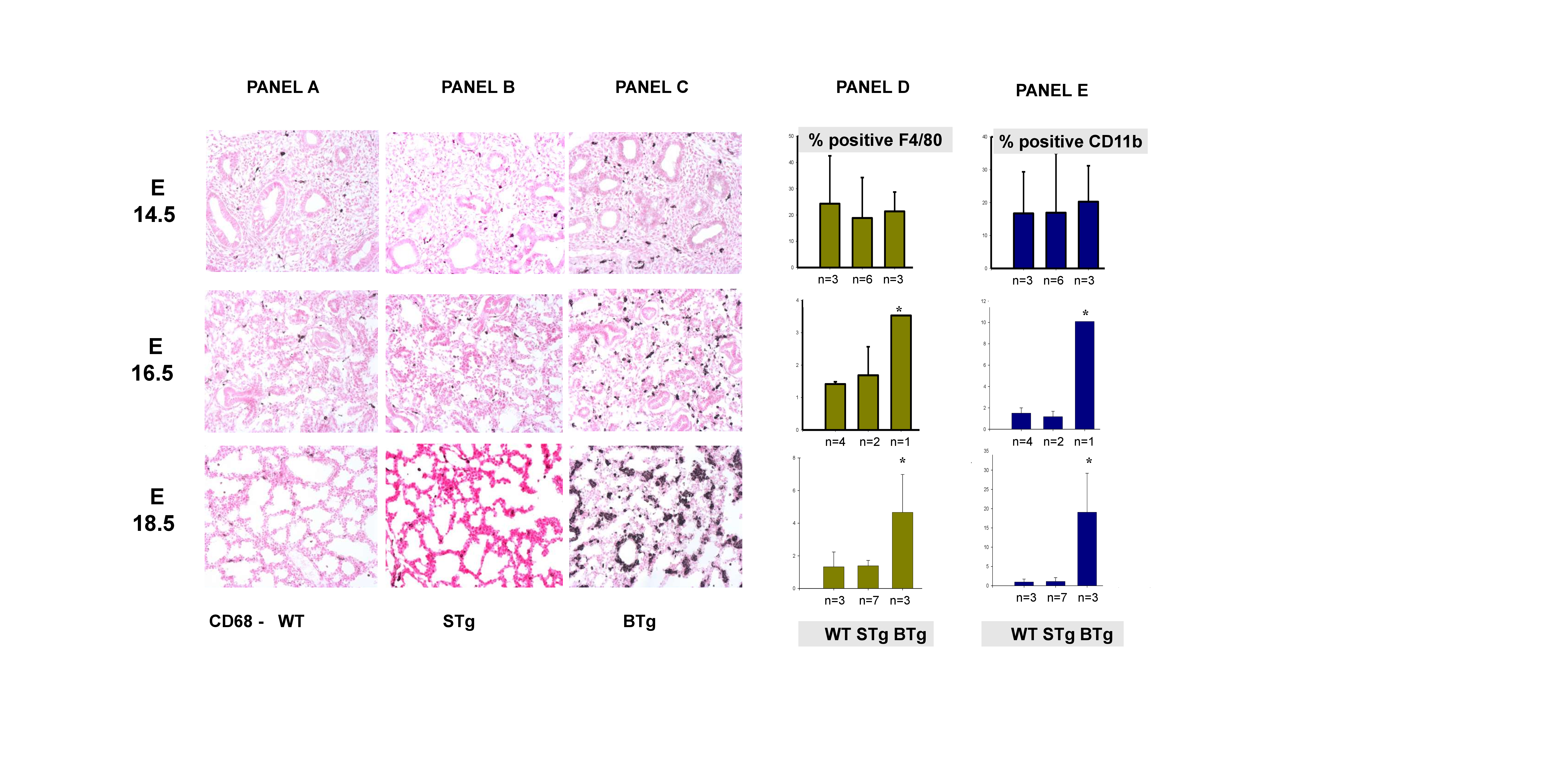

Supplement: Supplementary Figure 1 — Immunohistology and flow cytometry quantification of lung macrophages using CD68, F4/80 and CD11b markers in mice with constitutive and inducible GM-CSF backgrounds. (A–E) Represent results from E14.5 mice on regular chow and from mice who were on Dox at E16.5 and E18.5. There were no differences in CD68, F4/80 and CD11b positive macrophages between WT and STg at E14.5, E16.5 or E18.5. However, significantly more of these macrophages were present in BTg on Dox at E16.5 and E18.5. WT, wild type; STg, single transgenic; BTg, bitransgenic; *p < 0.05 vs. controls. [file Image_1.TIFF]

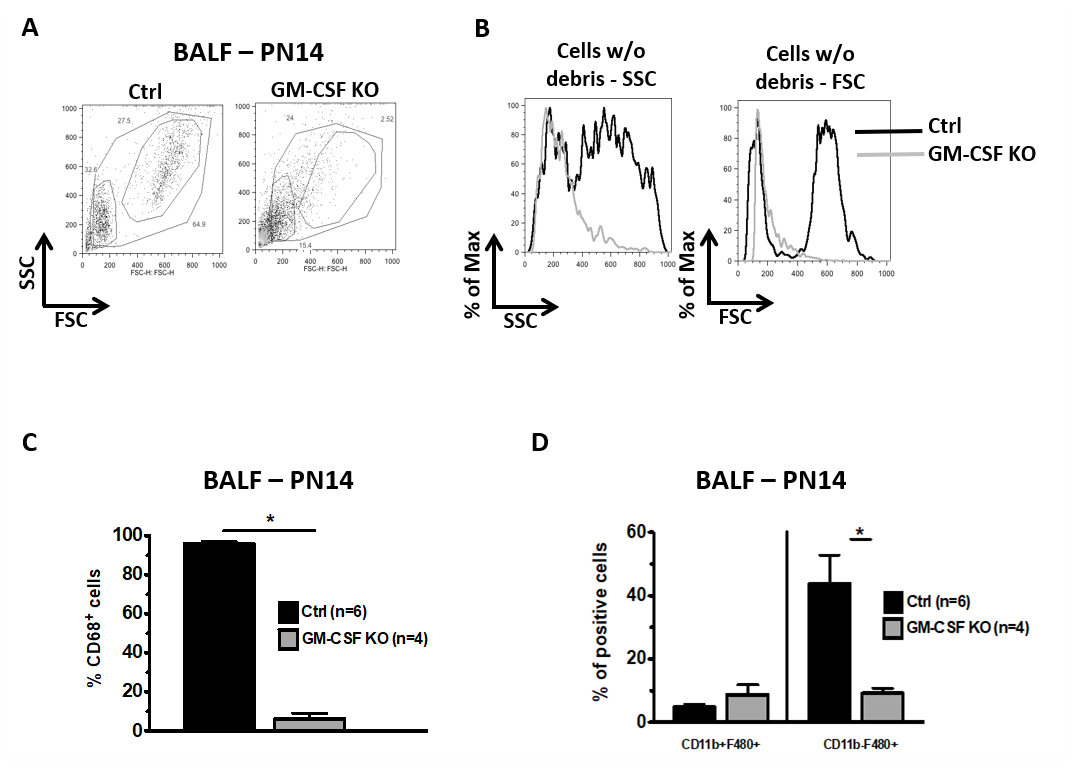

Supplement: Supplementary Figure 2 — Immunophenotyping of BALF cells for monocyte/macrophage surface markers in control and GM-CSF KO neonatal mice. Flow cytometry was performed on BALF cells from control and GM-CSF KO neonatal mice at PN14. (A) Representative dot plots and (B) histogram of the forward-scatter (FSC) and side-scatter (SSC) properties of BALF cells. (C) Quantitation of CD68+ cells and (D) CD11b+ and F4/80+ cells. In contrast to the subtle differences between cell populations in lung homogenates (Figure 4J), BALF from GM-CSF KO mice had strikingly lower CD68+ cells, and lower CD11b− F4/80+ cells compared to controls (*p < 0.05 vs. controls). [file Image_2.tif]
